# 6,6′-Dieth­oxy-2,2′-[4-methyl-1,2-phenyl­enebis(nitrilo­methanylyl­idene)]diphenol aceto­nitrile monosolvate

**DOI:** 10.1107/S1600536813028845

**Published:** 2013-10-26

**Authors:** Lei Li, Suyuan Zeng

**Affiliations:** a Liaocheng University, Department of Chemistry, Liaocheng, Shandong Province 252059, People’s Republic of China

## Abstract

The title solvated Schiff base compound, C_25_H_26_N_2_O_4_·CH_3_CN, possesses an O_2_N_2_ donor set affording a potentially tetra­dentate metal complex ligand. The central ring makes dihedral angles of 6.7 (3) and 48.4 (2)° with the pendant rings. Intra­molecular N—H⋯O hydrogen-bonding inter­actions are observed.

## Related literature

For background to the properties of tetra­dentate Schiff-base ligands with O_2_N_2_ donor sets, see Zhang *et al.* (2009 ▶); Nayka *et al.* (2006 ▶). For related crystal structures, see Liu *et al.* (2006 ▶); Kargar *et al.* (2009 ▶). 
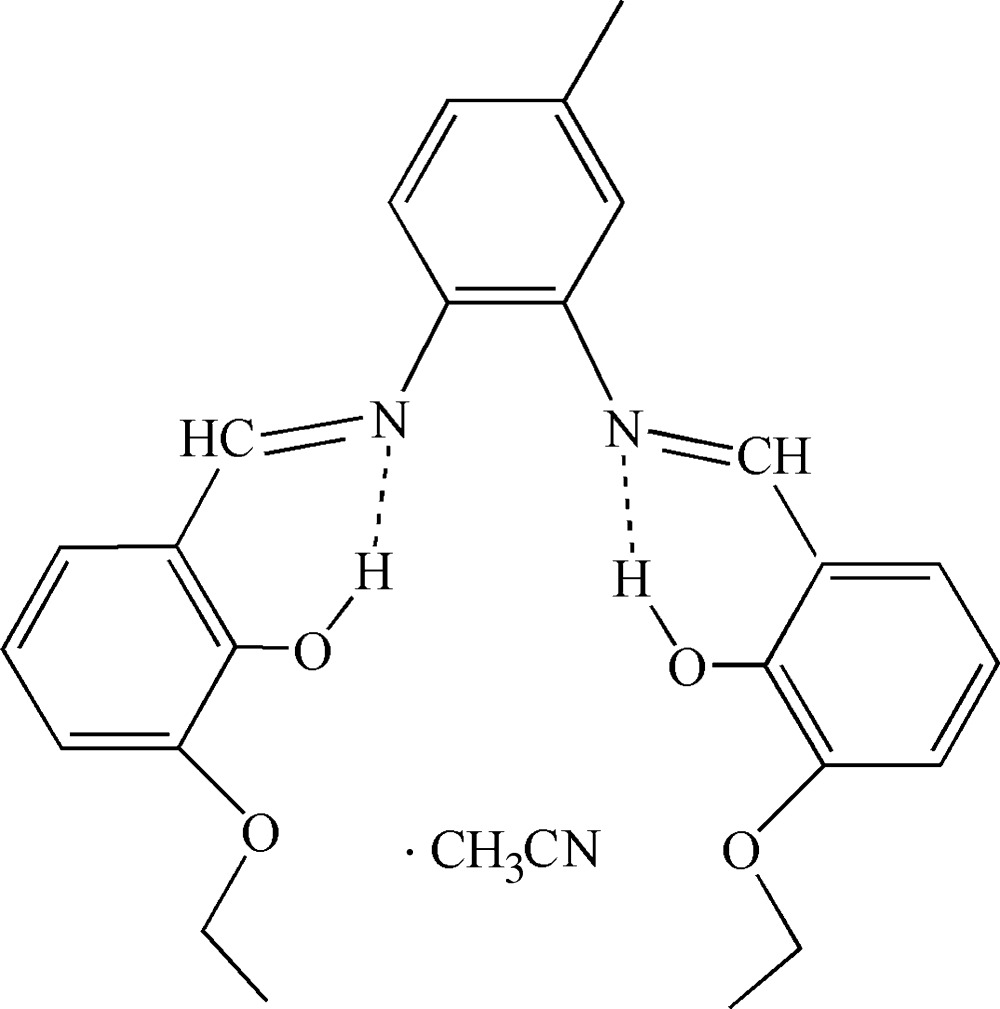


## Experimental

### 

#### Crystal data

C_25_H_26_N_2_O_4_·C_2_H_3_N
*M* *_r_* = 459.53
Monoclinic, 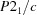

*a* = 11.580 (3) Å
*b* = 24.999 (7) Å
*c* = 8.995 (3) Å
β = 106.891 (6)°
*V* = 2491.7 (12) Å^3^
*Z* = 4
Mo *K*α radiation
μ = 0.08 mm^−1^
*T* = 293 K
0.17 × 0.11 × 0.09 mm

#### Data collection

Bruker APEXII CCD area-detector diffractometer
Absorption correction: multi-scan (*SADABS*; Sheldrick, 2008*a* ▶) *T*_min_ = 0.986, *T*_max_ = 0.993
12206 measured reflections
4387 independent reflections
2242 reflections with *I* > 2σ(*I*)
*R*_int_ = 0.122

#### Refinement

*R*[*F* ^2^ > 2σ(*F* ^2^)] = 0.061
*wR*(*F* ^2^) = 0.171
*S* = 0.91
4387 reflections
313 parameters
H-atom parameters constrained
Δρ_max_ = 0.14 e Å^−3^
Δρ_min_ = −0.17 e Å^−3^

### 

Data collection: *APEX2* (Bruker, 2004 ▶); cell refinement: *SAINT-Plus* (Bruker, 2001 ▶); data reduction: *SAINT-Plus*; program(s) used to solve structure: *SHELXS97* (Sheldrick, 2008*b* ▶; program(s) used to refine structure: *SHELXL97* (Sheldrick, 2008*b* ▶); molecular graphics: *SHELXTL* (Sheldrick, 2008*b* ▶); software used to prepare material for publication: *SHELXTL*.

## Supplementary Material

Crystal structure: contains datablock(s) I, global. DOI: 10.1107/S1600536813028845/hg5352sup1.cif

Structure factors: contains datablock(s) I. DOI: 10.1107/S1600536813028845/hg5352Isup2.hkl

Additional supplementary materials:  crystallographic information; 3D view; checkCIF report

## Figures and Tables

**Table 1:** Hydrogen-bond geometry (Å, °)

*D*—H⋯*A*	*D*—H	H⋯*A*	*D*⋯*A*	*D*—H⋯*A*
O1—H1⋯N1	0.82	1.90	2.610 (5)	145
O2—H2⋯N2	0.82	1.91	2.605 (5)	142

**Fig. 1.**
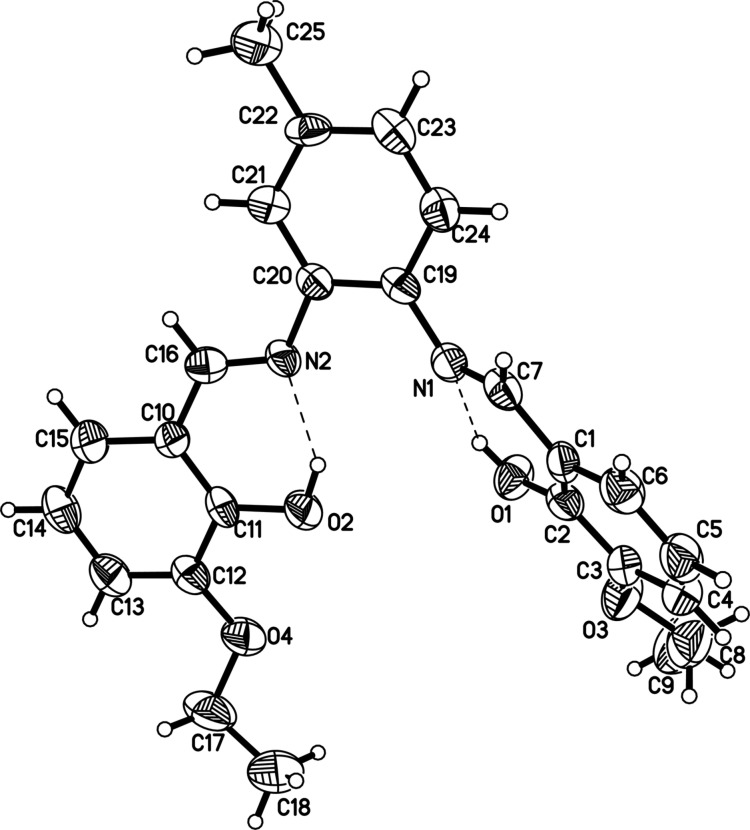
The structure of the title compound with the atom-labelling scheme. Displacement ellipsoids are drawn at the 30% probability level. The solvent molecule has been omitted for clarity.

## supplementary crystallographic information

### 1. Comment

During the past several decades, tetradentate Schiff-base ligands with O_2_N_2_ donor sets have been studied intensively, partially due to the interesting magnetic properties observed for their metal complexes (Zhang *et al.*, 2009; Nayak *etal.*. Herein, we present the crystal structure of a new tetradentate Schiff base ligand *N*,*N*'-Bis(2-hydroxy-3-ethoxybenzylidene)-4-methyl-1,2-phenylenediamine as its acetonitrile solvate.

As shown in Figure 1, the title compound possesses a O_2_N_2_ donor set affording the potentially tetradentate ligand. The imide bond lengths 1.296 (5)Å for N1—C7 and 1.269 (5)Å for N2—C16 are slightly shorter than that of related Schiff-base ligands *N*,*N*'-Bis(2-hydroxy-3-methoxybenzylidene)-1,2- phenylenediamine (Liu, *et al.*, 2006) and 6,6'-Diethoxy-2,2'- [4,5-dimethyl-*o*-phenylenebis(nitrilomethylidyne)]diphenol (Kargar, *et al.* 2009). In this compound, two relative strong O-H···N intramolecular bonds, O1-H1···N1 and O2-H2···N2 are observed (Table 1).

### 2. Experimental

The Schiff base ligand was prepared by condensation 4-methyl-1,2-phenylenediamine (10 mmol, 1.22 g) and 2-hydroxy-3-ethoxybenzaldehyde (20 mmol, 3.32 g) in a mixture of ethanol and acetonitrile(1:1). The mixture formed was allowed to partial evaporate in air for about one week to produce crystals suitable for X-ray diffraction.

### 3. Refinement

All the H atoms bonded to C atoms were placed using the HFIX commands in *SHELXL-97*, with C—H distances of 0.93, 0.96, 0.97Å, and were allowed for as riding atoms with *U*_iso_(H) = 1.2*U*_eq_(C) and *U*_iso_(H) = 1.5*U*_eq_(C) (methyl) respectively. The hydroxyl protons were located from difference Fourier maps with the O—H bond length restrained to 0.82 Å and was allowed for as riding atoms with *U*_iso_(H) = 1.2*U*_eq_(O).

### Crystal data

**Table:**

C_25_H_26_N_2_O_4_·C_2_H_3_N	*F*(000) = 976
*M**_r_* = 459.53	*D*_x_ = 1.225 Mg m^−^^3^
Monoclinic, *P*2_1_/*c*	Mo *K*α radiation, λ = 0.71073 Å
Hall symbol: -P 2ybc	Cell parameters from 1236 reflections
*a* = 11.580 (3) Å	θ = 2.3–26.3°
*b* = 24.999 (7) Å	µ = 0.08 mm^−^^1^
*c* = 8.995 (3) Å	*T* = 293 K
β = 106.891 (6)°	Block, orange
*V* = 2491.7 (12) Å^3^	0.17 × 0.11 × 0.09 mm
*Z* = 4	

### Data collection

**Table:**

Bruker APEXII CCD area-detector diffractometer	4387 independent reflections
Radiation source: fine-focus sealed tube	2242 reflections with *I* > 2σ(*I*)
Graphite monochromator	*R*_int_ = 0.122
φ and ω scans	θ_max_ = 25.0°, θ_min_ = 1.8°
Absorption correction: multi-scan (*SADABS*; Sheldrick, 2008*a*)	*h* = −13→12
*T*_min_ = 0.986, *T*_max_ = 0.993	*k* = −29→29
12206 measured reflections	*l* = −4→10

### Refinement

**Table:**

Refinement on *F*^2^	Primary atom site location: structure-invariant direct methods
Least-squares matrix: full	Secondary atom site location: difference Fourier map
*R*[*F*^2^ > 2σ(*F*^2^)] = 0.061	Hydrogen site location: inferred from neighbouring sites
*wR*(*F*^2^) = 0.171	H-atom parameters constrained
*S* = 0.91	*w* = 1/[σ^2^(*F*_o_^2^) + (0.0517*P*)^2^] where *P* = (*F*_o_^2^ + 2*F*_c_^2^)/3
4387 reflections	(Δ/σ)_max_ = 0.011
313 parameters	Δρ_max_ = 0.14 e Å^−^^3^
0 restraints	Δρ_min_ = −0.17 e Å^−^^3^

### Special details

**Table:**

Geometry. All e.s.d.'s (except the e.s.d. in the dihedral angle between two l.s. planes) are estimated using the full covariance matrix. The cell e.s.d.'s are taken into account individually in the estimation of e.s.d.'s in distances, angles and torsion angles; correlations between e.s.d.'s in cell parameters are only used when they are defined by crystal symmetry. An approximate (isotropic) treatment of cell e.s.d.'s is used for estimating e.s.d.'s involving l.s. planes.
Refinement. Refinement of *F*^2^ against ALL reflections. The weighted *R*-factor *wR* and goodness of fit *S* are based on *F*^2^, conventional *R*-factors *R* are based on *F*, with *F* set to zero for negative *F*^2^. The threshold expression of *F*^2^ > σ(*F*^2^) is used only for calculating *R*-factors(gt) *etc*. and is not relevant to the choice of reflections for refinement. *R*-factors based on *F*^2^ are statistically about twice as large as those based on *F*, and *R*- factors based on ALL data will be even larger.

### Fractional atomic coordinates and isotropic or equivalent isotropic displacement parameters (Å^2^)

**Table:**

	*x*	*y*	*z*	*U*_iso_*/*U*_eq_	
O1	0.7285 (3)	0.11495 (14)	0.4282 (4)	0.0813 (10)	
H1	0.7486	0.0945	0.3688	0.122*	
O2	0.5747 (3)	0.09311 (14)	0.0478 (4)	0.0904 (11)	
H2	0.6313	0.0732	0.0889	0.136*	
O3	0.6804 (4)	0.19431 (15)	0.5910 (4)	0.1040 (13)	
O4	0.3638 (3)	0.13571 (16)	−0.0824 (4)	0.1035 (13)	
N1	0.8803 (3)	0.06746 (18)	0.3066 (4)	0.0636 (11)	
N2	0.7374 (3)	0.02192 (14)	0.0410 (4)	0.0625 (11)	
N3	0.2332 (5)	0.1357 (3)	0.3047 (7)	0.163 (3)	
C1	0.9221 (5)	0.1516 (2)	0.4400 (5)	0.0645 (13)	
C2	0.8122 (5)	0.1537 (2)	0.4743 (6)	0.0672 (13)	
C3	0.7888 (6)	0.1968 (2)	0.5600 (6)	0.0740 (15)	
C4	0.8739 (6)	0.2366 (2)	0.6093 (6)	0.0879 (17)	
H4	0.8591	0.2649	0.6682	0.105*	
C5	0.9807 (6)	0.2343 (3)	0.5712 (7)	0.0958 (19)	
H5	1.0361	0.2621	0.6017	0.115*	
C6	1.0070 (5)	0.1926 (2)	0.4900 (6)	0.0831 (16)	
H6	1.0806	0.1912	0.4679	0.100*	
C7	0.9526 (4)	0.1072 (2)	0.3588 (5)	0.0668 (14)	
H7	1.0283	0.1068	0.3424	0.080*	
C8	0.6624 (6)	0.2277 (3)	0.7098 (7)	0.136 (2)	
H8A	0.6499	0.2643	0.6729	0.163*	
H8B	0.7333	0.2268	0.7995	0.163*	
C9	0.5578 (7)	0.2092 (3)	0.7534 (9)	0.161 (3)	
H9A	0.4886	0.2086	0.6632	0.242*	
H9B	0.5428	0.2330	0.8296	0.242*	
H9C	0.5727	0.1738	0.7961	0.242*	
C10	0.3962 (5)	0.0948 (2)	−0.1623 (7)	0.0775 (15)	
C11	0.5093 (4)	0.0717 (2)	−0.0890 (6)	0.0646 (13)	
C12	0.5510 (4)	0.0303 (2)	−0.1607 (6)	0.0676 (14)	
C13	0.4802 (5)	0.0110 (2)	−0.3033 (6)	0.1016 (19)	
H13	0.5082	−0.0169	−0.3521	0.122*	
C14	0.3700 (5)	0.0329 (3)	−0.3710 (7)	0.1034 (19)	
H14	0.3226	0.0196	−0.4656	0.124*	
C15	0.3277 (5)	0.0744 (2)	−0.3020 (7)	0.0897 (17)	
H15	0.2520	0.0889	−0.3501	0.108*	
C16	0.6679 (5)	0.00675 (19)	−0.0889 (6)	0.0761 (15)	
H16	0.6935	−0.0210	−0.1406	0.091*	
C17	0.2618 (5)	0.1673 (2)	−0.1656 (7)	0.119 (2)	
H17A	0.1879	0.1467	−0.1840	0.142*	
H17B	0.2705	0.1785	−0.2650	0.142*	
C18	0.2581 (6)	0.2146 (3)	−0.0671 (8)	0.164 (3)	
H18A	0.2607	0.2029	0.0355	0.246*	
H18B	0.1851	0.2343	−0.1116	0.246*	
H18C	0.3264	0.2371	−0.0615	0.246*	
C19	0.9238 (4)	0.0225 (2)	0.2448 (6)	0.0616 (13)	
C20	0.8512 (4)	−0.0026 (2)	0.1097 (6)	0.0606 (13)	
C21	0.8941 (4)	−0.0476 (2)	0.0549 (5)	0.0726 (14)	
H21	0.8461	−0.0644	−0.0338	0.087*	
C22	1.0074 (5)	−0.0684 (2)	0.1290 (6)	0.0714 (15)	
C23	1.0780 (5)	−0.0442 (2)	0.2597 (7)	0.0825 (16)	
H23	1.1537	−0.0582	0.3102	0.099*	
C24	1.0373 (4)	0.0007 (2)	0.3168 (5)	0.0755 (15)	
H24	1.0866	0.0171	0.4056	0.091*	
C25	1.0510 (4)	−0.1184 (2)	0.0656 (6)	0.1002 (18)	
H25A	1.1363	−0.1158	0.0802	0.150*	
H25B	1.0097	−0.1218	−0.0432	0.150*	
H25C	1.0345	−0.1493	0.1197	0.150*	
C26	0.4564 (5)	0.1408 (2)	0.3195 (7)	0.142 (3)	
H26A	0.4775	0.1771	0.3037	0.213*	
H26B	0.4710	0.1184	0.2402	0.213*	
H26C	0.5046	0.1287	0.4196	0.213*	
C27	0.3299 (7)	0.1382 (2)	0.3121 (7)	0.107 (2)	

### Atomic displacement parameters (Å^2^)

**Table:**

	*U*^11^	*U*^22^	*U*^33^	*U*^12^	*U*^13^	*U*^23^
O1	0.062 (2)	0.089 (3)	0.090 (3)	−0.013 (2)	0.0185 (18)	−0.025 (2)
O2	0.078 (3)	0.113 (3)	0.068 (2)	0.030 (2)	0.001 (2)	−0.010 (2)
O3	0.091 (3)	0.121 (3)	0.103 (3)	−0.009 (2)	0.032 (2)	−0.045 (2)
O4	0.088 (3)	0.118 (3)	0.095 (3)	0.047 (2)	0.013 (2)	0.004 (3)
N1	0.059 (3)	0.070 (3)	0.060 (3)	0.003 (2)	0.013 (2)	0.004 (2)
N2	0.052 (3)	0.068 (3)	0.061 (3)	0.004 (2)	0.006 (2)	0.004 (2)
N3	0.094 (4)	0.244 (7)	0.155 (5)	−0.029 (5)	0.045 (5)	0.001 (5)
C1	0.073 (4)	0.066 (4)	0.046 (3)	−0.008 (3)	0.006 (3)	−0.002 (3)
C2	0.059 (4)	0.072 (4)	0.063 (3)	0.000 (3)	0.006 (3)	0.006 (3)
C3	0.079 (4)	0.080 (4)	0.055 (4)	0.008 (4)	0.007 (3)	−0.008 (3)
C4	0.106 (5)	0.069 (4)	0.074 (4)	−0.003 (4)	0.003 (4)	−0.011 (3)
C5	0.085 (5)	0.090 (5)	0.096 (5)	−0.025 (4)	0.000 (4)	0.009 (4)
C6	0.082 (4)	0.083 (4)	0.075 (4)	−0.008 (4)	0.008 (3)	0.012 (3)
C7	0.057 (3)	0.089 (4)	0.052 (3)	−0.001 (3)	0.011 (3)	0.008 (3)
C8	0.141 (6)	0.161 (7)	0.106 (5)	0.008 (6)	0.035 (5)	−0.044 (5)
C9	0.178 (7)	0.151 (7)	0.192 (7)	−0.023 (5)	0.112 (6)	−0.049 (5)
C10	0.065 (4)	0.089 (4)	0.074 (4)	0.007 (3)	0.014 (3)	0.012 (4)
C11	0.053 (3)	0.081 (4)	0.053 (4)	−0.002 (3)	0.005 (3)	0.006 (3)
C12	0.065 (4)	0.073 (4)	0.060 (4)	0.006 (3)	0.011 (3)	−0.002 (3)
C13	0.093 (4)	0.110 (5)	0.077 (4)	0.016 (4)	−0.015 (4)	−0.018 (4)
C14	0.088 (5)	0.116 (5)	0.080 (4)	0.006 (4)	−0.016 (4)	−0.012 (4)
C15	0.069 (4)	0.110 (5)	0.075 (5)	0.002 (4)	−0.003 (4)	0.017 (4)
C16	0.070 (4)	0.085 (4)	0.070 (4)	0.015 (3)	0.015 (3)	−0.009 (3)
C17	0.099 (5)	0.125 (6)	0.121 (5)	0.047 (4)	0.014 (4)	0.035 (5)
C18	0.164 (7)	0.154 (7)	0.158 (7)	0.094 (5)	0.023 (5)	−0.004 (6)
C19	0.050 (3)	0.083 (4)	0.052 (3)	0.009 (3)	0.015 (3)	0.012 (3)
C20	0.059 (3)	0.066 (4)	0.059 (4)	0.009 (3)	0.021 (3)	0.011 (3)
C21	0.073 (4)	0.067 (4)	0.077 (4)	0.003 (3)	0.020 (3)	0.002 (3)
C22	0.082 (4)	0.067 (4)	0.078 (4)	0.027 (3)	0.043 (3)	0.021 (3)
C23	0.070 (4)	0.100 (5)	0.072 (4)	0.016 (4)	0.012 (3)	0.022 (4)
C24	0.058 (4)	0.101 (5)	0.067 (4)	0.004 (3)	0.017 (3)	0.006 (3)
C25	0.095 (4)	0.100 (4)	0.105 (4)	0.023 (4)	0.027 (4)	0.014 (4)
C26	0.061 (4)	0.164 (6)	0.192 (7)	−0.002 (4)	0.024 (4)	−0.054 (5)
C27	0.099 (5)	0.119 (5)	0.103 (5)	−0.009 (5)	0.029 (5)	−0.013 (4)

### Geometric parameters (Å, º)

**Table:**

O1—C2	1.347 (5)	C11—C12	1.381 (6)
O1—H1	0.8200	C12—C13	1.393 (6)
O2—C11	1.353 (5)	C12—C16	1.444 (6)
O2—H2	0.8200	C13—C14	1.361 (6)
O3—C3	1.364 (6)	C13—H13	0.9300
O3—C8	1.418 (6)	C14—C15	1.369 (6)
O4—C10	1.364 (5)	C14—H14	0.9300
O4—C17	1.437 (5)	C15—H15	0.9300
N1—C7	1.296 (5)	C16—H16	0.9300
N1—C19	1.411 (5)	C17—C18	1.486 (7)
N2—C16	1.269 (5)	C17—H17A	0.9700
N2—C20	1.421 (5)	C17—H17B	0.9700
N3—C27	1.105 (6)	C18—H18A	0.9600
C1—C2	1.394 (6)	C18—H18B	0.9600
C1—C6	1.400 (6)	C18—H18C	0.9600
C1—C7	1.428 (6)	C19—C24	1.396 (6)
C2—C3	1.397 (6)	C19—C20	1.407 (6)
C3—C4	1.379 (6)	C20—C21	1.378 (5)
C4—C5	1.377 (6)	C21—C22	1.388 (6)
C4—H4	0.9300	C21—H21	0.9300
C5—C6	1.359 (6)	C22—C23	1.362 (6)
C5—H5	0.9300	C22—C25	1.521 (6)
C6—H6	0.9300	C23—C24	1.375 (6)
C7—H7	0.9300	C23—H23	0.9300
C8—C9	1.453 (7)	C24—H24	0.9300
C8—H8A	0.9700	C25—H25A	0.9600
C8—H8B	0.9700	C25—H25B	0.9600
C9—H9A	0.9600	C25—H25C	0.9600
C9—H9B	0.9600	C26—C27	1.448 (7)
C9—H9C	0.9600	C26—H26A	0.9600
C10—C15	1.375 (6)	C26—H26B	0.9600
C10—C11	1.407 (6)	C26—H26C	0.9600
			
C2—O1—H1	109.5	C13—C14—H14	119.5
C11—O2—H2	109.5	C15—C14—H14	119.5
C3—O3—C8	118.2 (5)	C14—C15—C10	120.5 (5)
C10—O4—C17	116.6 (4)	C14—C15—H15	119.7
C7—N1—C19	119.2 (4)	C10—C15—H15	119.7
C16—N2—C20	122.1 (4)	N2—C16—C12	123.5 (5)
C2—C1—C6	120.2 (5)	N2—C16—H16	118.3
C2—C1—C7	121.3 (5)	C12—C16—H16	118.3
C6—C1—C7	118.5 (6)	O4—C17—C18	106.7 (5)
O1—C2—C1	121.8 (5)	O4—C17—H17A	110.4
O1—C2—C3	119.1 (5)	C18—C17—H17A	110.4
C1—C2—C3	119.1 (5)	O4—C17—H17B	110.4
O3—C3—C4	125.5 (6)	C18—C17—H17B	110.4
O3—C3—C2	114.6 (5)	H17A—C17—H17B	108.6
C4—C3—C2	119.9 (6)	C17—C18—H18A	109.5
C5—C4—C3	120.0 (6)	C17—C18—H18B	109.5
C5—C4—H4	120.0	H18A—C18—H18B	109.5
C3—C4—H4	120.0	C17—C18—H18C	109.5
C6—C5—C4	121.5 (6)	H18A—C18—H18C	109.5
C6—C5—H5	119.3	H18B—C18—H18C	109.5
C4—C5—H5	119.3	C24—C19—C20	118.3 (5)
C5—C6—C1	119.3 (6)	C24—C19—N1	121.5 (5)
C5—C6—H6	120.4	C20—C19—N1	120.2 (4)
C1—C6—H6	120.4	C21—C20—C19	119.0 (5)
N1—C7—C1	123.5 (5)	C21—C20—N2	125.5 (5)
N1—C7—H7	118.3	C19—C20—N2	115.5 (5)
C1—C7—H7	118.3	C20—C21—C22	121.5 (5)
O3—C8—C9	109.5 (6)	C20—C21—H21	119.2
O3—C8—H8A	109.8	C22—C21—H21	119.2
C9—C8—H8A	109.8	C23—C22—C21	119.6 (5)
O3—C8—H8B	109.8	C23—C22—C25	120.4 (5)
C9—C8—H8B	109.8	C21—C22—C25	119.9 (6)
H8A—C8—H8B	108.2	C22—C23—C24	120.0 (5)
C8—C9—H9A	109.5	C22—C23—H23	120.0
C8—C9—H9B	109.5	C24—C23—H23	120.0
H9A—C9—H9B	109.5	C23—C24—C19	121.5 (5)
C8—C9—H9C	109.5	C23—C24—H24	119.2
H9A—C9—H9C	109.5	C19—C24—H24	119.2
H9B—C9—H9C	109.5	C22—C25—H25A	109.5
O4—C10—C15	125.9 (5)	C22—C25—H25B	109.5
O4—C10—C11	114.9 (5)	H25A—C25—H25B	109.5
C15—C10—C11	119.2 (6)	C22—C25—H25C	109.5
O2—C11—C12	122.8 (5)	H25A—C25—H25C	109.5
O2—C11—C10	117.6 (5)	H25B—C25—H25C	109.5
C12—C11—C10	119.6 (5)	C27—C26—H26A	109.5
C11—C12—C13	119.9 (5)	C27—C26—H26B	109.5
C11—C12—C16	120.4 (5)	H26A—C26—H26B	109.5
C13—C12—C16	119.7 (5)	C27—C26—H26C	109.5
C14—C13—C12	119.8 (5)	H26A—C26—H26C	109.5
C14—C13—H13	120.1	H26B—C26—H26C	109.5
C12—C13—H13	120.1	N3—C27—C26	179.0 (8)
C13—C14—C15	121.0 (6)		

### Hydrogen-bond geometry (Å, º)

**Table:**

*D*—H···*A*	*D*—H	H···*A*	*D*···*A*	*D*—H···*A*
O1—H1···N1	0.82	1.90	2.610 (5)	145
O2—H2···N2	0.82	1.91	2.605 (5)	142
